# Willingness to pay for certification and labeling of chicken meat in the Mymensingh District of Bangladesh

**DOI:** 10.5455/javar.2024.k829

**Published:** 2024-09-30

**Authors:** Mahbuba Akther Mishu, Sourav Mohan Saha, Md. Masudul Haque Prodhan, Md. Mostafijur Rahman, Md. Akhtaruzzaman Khan

**Affiliations:** 1 Department of Agricultural Finance, Co-operatives and Banking, Khulna Agricultural University, Khulna, Bangladesh; 2 Policy Research Institute of Bangladesh, Dhaka, Bangladesh; 3 Economics Discipline, Khulna University, Khulna, Bangladesh; 4 Department of Agricultural Finance and Banking, Faculty of Agricultural Economics and Rural Sociology, Bangladesh Agricultural University, Mymensingh, Bangladesh; † These authors contributed equally to this paper

**Keywords:** Food safety, certification and labeling, consumer preferences, poultry industry

## Abstract

**Objective:**

This study explores consumers’ willingness to pay (WTP) for labeled and certified chicken meat in Mymensingh district, examining the key socioeconomic and demographic factors that shape these preferences.

**Materials and Method:**

Data were gathered through structured interviews with 300 participants from diverse urban and rural demographics, encompassing various occupational groups. The analysis employed logistic regression models to examine the determinants of WTP for labeling and certification, with a focus on variables such as income, education, health perception, environmental awareness, dependency ratio, and market distance.

**Results:**

The study found that a significant proportion of consumers preferred labeled (64%) and certified (71%) broiler meat. Strong links between higher income and education levels and increased WTP suggest that wealthier and more literate consumers are more likely to invest in food safety measures. Positive health and environmental perceptions also played a role, with more conscious consumers willing to pay a premium for labeled and certified products. On the other hand, a higher dependency ratio and greater market distance were associated with lower WTP, underscoring the impact of socioeconomic constraints and accessibility issues on consumer choices.

**Conclusion:**

Introducing labeling and certification systems could strengthen consumer trust and enhance public health, offering substantial benefits to both the poultry industry and the broader economy in Bangladesh.

## Introduction

The poultry industry is persistently accelerating and becoming more industrialized in numerous regions worldwide, resulting from an expanding population [1], rising per capita income [2], and increased urbanization. In response to increasing global demand, the production of chicken meat had a substantial surge [3] to 133 million metric tons in 2020, an increase from 9 million metric tons in 1961, accompanied by egg production, which witnessed a significant promotion, soaring from 15 million metric tons to 93 million metric tons during the same period [4]. Most of this progress has occurred in Asia, where production has nearly quadrupled [1,4]. In Europe, beef and pork consumption has decreased due to health and environmental sustainability concerns [5]. Chicken meat production is undertaken by around 80% of rural families in developing nations [3,5]. In developing nations, the quality of food products is challenged by various problems, such as the presence of multiple antibiotics in food products [3], which can be associated with the food industry’s inadequate assessment of food security risks [6], governments, and consumers [7].

The poultry industry holds significant importance and demonstrates advanced development within Bangladesh’s livestock sector [8], attracting attention from local entrepreneurs and foreign investors [2]. The demand for chicken meat in Bangladesh has significantly increased over the past few years [1]. The livestock sector in Bangladesh plays an immense part in enhancing the economy of the country, assuring food and nutrition security, generating employment opportunities, and, importantly, reducing poverty [9]. The egg production in the fiscal year 2021–2022 amounted to 2,335.35 crores, which is 3.8 times higher than the egg production in the fiscal year 2010–2011, which was 607.85 crores. Additionally, the per capita egg availability reached 136.01 eggs per year, as reported by the Bureau of Economic Research in 2023. Food safety is a global issue because a significant proportion of the global population, around 1 in 10 people, suffers illness from consuming contaminated food [10]. Furthermore, the consequences of foodborne infections are severe, leading to an estimated annual death toll of 420,000 people [11]. So, the focus is on health effects, while foodborne diseases have significant economic impacts, in terms of products discarded, health care and treatment costs, inability to work, and the potential spread of epidemics.

The primary issues faced by a developing nation such as Bangladesh’s chicken meat consumption pertain to food security, food safety [12], and public health. In the past decade, there has been a growing concern regarding food safety due to the proliferation of contaminated and low-quality food products in the market [13]. That has resulted in a significant escalation of public health concerns. The certification and labeling of chicken meat play a crucial role in safeguarding consumer well-being and conveying essential information regarding product quality, source, and production methods [10,14,15]. The Bangladesh Food Safety Authority is working on the execution of a strategy plan that focuses on the establishment of a certification system tailored to domestically produced nutritious food products [1]. The Japanese Agricultural Standards establish the certification of sustainable chicken meat in Japan. These standards cover a variety of requirements, including domestic chicken breeds, domestic feed rice use, chicken manure recycling practices, and adherence to animal welfare standards [15].

Bangladesh lacks regulations regarding animal slaughter and sells poultry meat without any labels. The open marketplace in this country slaughters almost all poultry without maintaining any hygiene [16]. Furthermore, there are no pre-slaughter and post-slaughter inspections to guarantee safety against any pathogen or contamination [17]. The instruments used for slaughtering and defeathering are not sterilized, and butchers do not even clean their hands between two consecutive slaughters [18]. Following the processing of poultry, butchers typically pack the meat in a polythene bag and distribute it to the customers. These unhygienic practices easily transmit different diseases from sick birds to healthy ones [19]. Although there are several existing laws related to meat slaughter and safety formulated in Bangladesh, such as the Animal Slaughter & Meat Quality Control Act 2011 and the Food Safety Act 2013, the basic procedures for safe slaughter and meat safety are not clearly stated in these regulations [17].

However, consumers’ lack of consciousness about food safety risks plays one of the integral roles in this situation [17]. Due to lower socioeconomic conditions and a lack of awareness, very few consumers in Bangladesh consider the approval of a regulatory authority when buying food items [19]. Financial status largely influences consumers’ choice of quality food [17].

Table 1 presents an extensive literature review on labeling and certification for safer food completed in this study. The growing global demand for chicken meat has led to a corresponding rise in the intricacy of the supply chain [3]. According to Maryasa and Linarti U [20] and Mandal and Khan [21], the lack of transparency throughout the supply chain leads to suspicions regarding the safety of food [13], the welfare of animals, and environmental sustainability [14]. Globally, the certification of chicken meat [1,5,15] holds significant importance [22,23], which ensures the safety [3,15,24] and quality of food products [15,25,26]. According to Nawi et al. [6], Pandanwangi et al. [3], Morone et al. [27], and Abbas et al. [7], assert that the certification of food items significantly influences consumer purchasing behavior. Furthermore, the implementation of certification and labeling systems allows industry stakeholders to visually demonstrate their devotion to safeguarding security [15], maintaining high standards of quality [7,26,28], and promoting sustainability [14]. Producers who adopt certification and labeling schemes have the opportunity to access certain markets that demonstrate a willingness to pay (WTP) premium for products that match their ethical or environmental values [10]. By integrating farmers into the certification and labeling system, producers should minimize the frequency of disease outbreaks, reduce the use of antibiotics [24], and ensure food safety and environmental sustainability. According to Nawi et al. [6], traceability systems in meat purchasing significantly impact consumer preferences and WTP, emphasizing the need for robust certification systems. Similarly, Abbas et al. [7] highlighted that consumer confidence in food safety is crucial in the post-COVID-19 era, further underscoring the importance of sustainable market environments and reliable certification systems.

**Table 1.** Literature matrix.

Research title	Study area	Major insights	Source
Consumers’ preferences and WTP for traceability systems in purchasing meat and meat products	Malaysia	57.7% of respondents are willing to pay a higher price for traceable meat and meat products The fact that consumers value quality, designated origin, production, and fair trade in meat and meat products demonstrates their preference for these attributes. Factors such as gender, income, Halal certificate, and transparency influence consumers’ WTP for traceability systems.	[8]
Does product certification matter? A review of mechanisms to influence customer loyalty in the poultry feed industry	Indonesia	The certification of feed has a significant impact on the perceived quality of products within the poultry feed industry. The poultry feed sector has a positive influence on customer loyalty as a result of consumer trust and happiness. The study demonstrated the necessity of enhancing awareness among farmers on the relevance of certification as a reliable indicator of product quality.	[4]
WTP for safe chicken meat in Bangladesh: a contingent valuation approach	Bangladesh	Consumers in Bangladesh have a high perception of health and environmental risks associated with conventional broiler meat consumption. The depicted average premium for WTP for safe chicken meat is BDT 39.87 per kg. There exists an accord among consumers on the pressing need for safe chicken meat that meets safety standards. Furthermore, customers have expressed a strong willingness to increase their consumption by approximately 36% if such meat becomes readily accessible in the local market.	[16]
Consumer WTP for chicken welfare attributes in Kenya	Kenya	The study reveals that there exists an appealing attitude among consumers in Kenya towards chicken meat that has been certified and labeled. They are willing to pay a premium for these characteristics, with the greatest WTP for the absence of growth hormones and the least WTP for poultry raised in confined systems.	[32]
Understanding consumers’ intentions to purchase clean label products: evidence from Taiwan	Taiwan	The product knowledge of consumers significantly impacts their desire to purchase and renewed interest in “clean label” products. This study provides insights into marketing channels, suggesting that the food business may improve customer confidence in certified labeling foods to increase purchase intention while executing effective strategies.	[33]
Consumers’ WTP for GLOBALG.A.P. certified chicken: empirical evidence from a consumer survey in Bangladesh	Bangladesh	Consumers residing in Bangladesh have a considerable preference and WTP a premium for certified chicken products. In Bangladesh, there exists an emerging consumer demand for certified fresh food, with consumers expressing a WTP a premium for such certified food products.	[1]
WTP for food labelling schemes in Vietnam: a choice experiment on water Spinach	Vietnam	Vietnamese consumers showed an inclination to be willing to pay additional costs for food labeling initiatives. The level of understanding and engagement about food labeling schemes did not have significant impacts on the perceived value of Vietnamese consumers.	[12]
European consumers’ WTP for red meat labelling attributes	Finland, France, Greece, Italy, Spain, Turkey, and the United Kingdom.	The attributes of origin and labeling of chicken meat were widely appreciated in the majority of countries. The increasing attention to social advantages and the adoption of “ethical” practices in food production have created an opportunity for red meat producers to gain a competitive edge through differentiation. Consumer preferences and WTP for health-related and ethical guarantees might provide valuable insights for red meat producers that penetrate the European market.	[5]
Sustainable market environment and consumer confidence in food safety in China after COVID-19: urban consumer perspectives	China	Consumer confidence in meat and domestically-produced infant formulas (DIF) is low in China. Quality certification, organic origin, and traceability are valued by consumers. Price as an indicator of high quality for low confidence consumers.	[9]

In Bangladesh, the poultry industry faces a growing need to address several key challenges, including preventing disease outbreaks [29,30] reducing environmental impact [12,31], promoting humane animal treatment [3,12] economic factors [8]. Certification and labeling schemes are crucial in addressing these challenges by establishing transparent and verifiable chicken production [3,34] processing and marketing criteria. A considerable cohort of scholars has identified the WTP for certified food products reflects variations [5,10,14,34] with a positive attitude towards certified transportation [3,7] and welfare labeling [34,35] but a negative preference towards the utilization of antibiotics in chicken production [36,29].

Despite the substantial role of poultry industries in Bangladesh, like other developing countries, in ensuring a significant number of employment opportunities and providing affordable protein, the clarified understanding of consumer behavior is still inchoate. Across the globe, food safety, quality assurance, and ethical sourcing promulgate the importance of certification and labeling. The consumers’ WTP for labeling and certification is measured from different aspects around the world in a varied methodological way, though there is a lack of suitable methods that closely simulate the real market scenarios. In this study, we deployed the single bounded contingent valuation method (CVM), which is particularly effective in accurately assessing the consumers’ WTP for labeling and certification of chicken meat by capturing binary response data indicative of consumer behavior in a hypothetical market structure that helps to mitigate the bias that may arise from direct elicitation of maximum WTP from overstating or understating respondents true preferences in approximating the decision-making process that consumers undertake in actual market circumstances. As a result, the study investigates Bangladesh’s WTP for chicken meat certification and labeling.

## Materials and Methods

### Sampling technique and data collection

This study selected *Sadar Upazila* of Mymensingh district as the study area because the city is home to one of the biggest wet marketplaces for chicken meat in Bangladesh, along with Dhaka and Chittagong City [37]. The population of the city is 577 thousand [38], and the required sample size with a 6% margin of error, assuming a 50% response distribution and a 95% confidence interval, is calculated at 267. Hence, this study was conducted among 300 consumers. To make the sample more representative, data was collected from 9 different categories of consumers, namely teachers, farmers, government service holders, businessmen, private service holders (private sector jobs), shopkeepers, day laborers, rickshaw-pullers, and others. Here, “others” category means spot consumer, i.e., data were collected at the broiler market and selected instantly irrespective of their job. An equal number of respondents (30) from each category were selected purposefully, and 60 respondents were from the category “others” since it represented all random consumers. Both urban and rural areas were covered by this study. Farmers’ and daily laborers’ data were collected from the village level; rickshaw pullers data was collected from peri-urban areas; and the rest of the data were collected from urban areas. The questionnaire was designed with information on consumers’ socio-economic characteristics, buying behavior, and WTP for labeling and certification of chicken meat. The draft survey schedule was pre-tested by interviewing a sample of consumers, and necessary modifications were made based on their feedback to align with the key objectives. The data were then collected through face-to-face interviews. Before the survey, each respondent was briefed on the concepts of labeling and certification of chicken meat. Qualitative data were converted to quantitative form when necessary.

### Empirical methodology

To accurately assess consumers’ WTP for labeling and certification of chicken meat, a method that closely simulates real market scenarios is essential. In this study, we employed the single-bounded CVM, which is particularly effective in capturing binary response data indicative of consumer behavior in hypothetical market settings [39].

CVM is a survey-based economic approach widely used to explore non-market resources, such as environmental goods or, in this case, the added value of food labeling and certification. In our study, the single-bounded CVM approach was adopted. This approach involves asking respondents a straightforward, binary question about their WTP and an additional amount for a specific attribute—in this instance, labeling and certification of chicken meat. By using a binary response format (“yes” or “no”), the CVM method helps in approximating the decision-making process that consumers undergo in actual market situations. This approach mitigates the bias that can arise from direct elicitation of maximum WTP, where respondents might overstate or understate their true preferences.

Respondents were presented with two key questions: Would you pay extra if chicken meat is properly labeled? And would you pay extra if chicken meat is certified by the government or a third-party authority? These questions were designed to elicit clear, binary responses, enabling a more straightforward analysis of consumers’ preferences and the factors influencing their decisions. The use of two separate questions also allows for the differentiation between the perceived value of labeling and certification, as these may be viewed differently by consumers.

To identify the determinants influencing consumers’ choices for labeling and certification of chicken meat, we applied a binary logistic regression model. This statistical method is appropriate for analyzing binary dependent variables, as it estimates the probability of a particular outcome (in this case, a “yes” or “no” response) based on one or more predictor variables. In the binary logit model, y is a binary variable indicating consumers’ preference for labeling and certification, which takes the value *y* = 1 when consumers would pay for labeling and certification of chicken meat and ‘0’ otherwise. The study employs the following empirical model:

where X_1_ = income (BDT), X_2_ = age (years), X_3_ = marital status (dummy), X_4_ = dependency ratio, X_5_ = market distance (km), X_6_ = health perception score, and X_7_ = environmental perception score.

We cannot directly explain the coefficients derived from logistic regression. Therefore, marginal effects (MEs) were calculated, which measure the impact on the probability of observing each of several outcomes rather than on a single conditional mean. ME are more meaningful and interpretable. Robust regression estimation was also employed to address the potential issue of heteroscedasticity.

## Results and Discussion

### Socioeconomic characteristics of consumers

**Figure 1.**
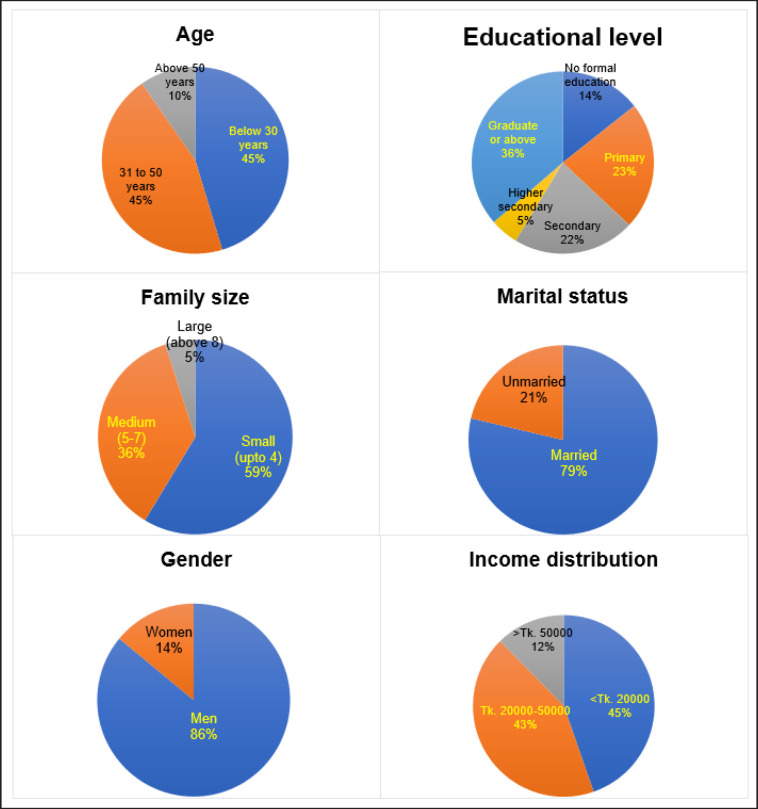
Demographic characteristics of respondents.

The demographic characteristics of respondents are presented in Figure 1. This section provides an overview of the age distribution, educational attainment, family size, marital status, gender, and income levels of the survey participants. About 45% of the respondents were from below 30 years and 30 to 50 years’ age groups each. Only about 10% of them are aged above 50 years. This suggests that the majority of respondents are within the economically active age groups, which might influence their purchasing behaviors and attitudes toward chicken meat certification and labeling. Most of the respondents (about 36%) had an education of at least graduation following secondary education level (about 22%). About 59% of them belonged to small families with up to four members. Marital status can be a significant factor in food purchasing patterns, as married individuals, particularly those with children, might prioritize food safety and quality more than unmarried individuals. The majority of the respondents in this study were married (about 79%), and only about 21% were unmarried. Most of them were males (about 86%) and only 14% were females. This gender disparity could be reflective of the cultural context in Bangladesh, where men are often the primary decision-makers in household purchases, including food items. This demographic characteristic needs to be considered when interpreting the results, as it might influence the generalizability of the findings to the broader population. About 45% of the respondents had an income of less than Tk. 20,000 per month, and about 43% of them had between Tk. 20,000 and Tk. 50,000. This indicates that a significant portion of the respondents are from lower to middle-income brackets. Income levels are a critical factor in determining WTP for premium products such as certified and labeled chicken meat, as higher income levels often correlate with greater discretionary spending capacity.

### Consumers’ preferences of labeling and certification for chicken meat

**Figure 2.**
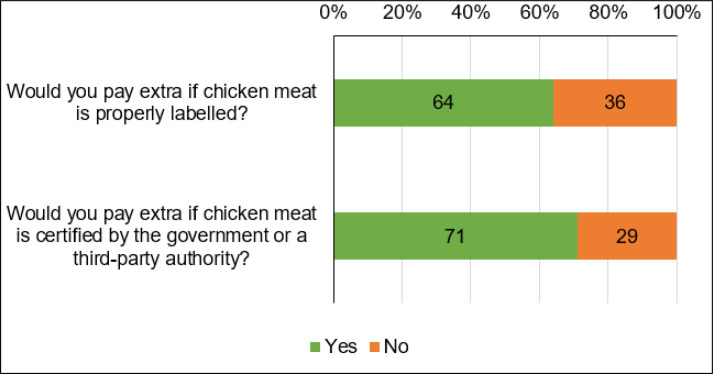
Consumers’ WTP for labeling and certification of chicken meat.

In this study, we first provided respondents with information about the general concept of labeling and certification to ensure they understood clearly before answering the survey questions. This step was crucial to avoid misconceptions influencing their WTP responses. When asked, “Would you pay extra if chicken meat is properly labeled?”, about 64% of the respondents answered positively (Fig. 2). This high percentage indicates a significant willingness among consumers to pay a premium for labeled chicken meat. Results suggest that a majority of consumers recognize the value of labeling, which typically includes information about the product’s origin, safety standards, and possibly nutritional content. In response to the question, “Would you pay extra if chicken meat is certified by the government or a third-party authority?”, about 71% of respondents agreed to pay extra for certified chicken meat.

This higher percentage compared to the WTP for labeling alone indicates an even stronger preference for certified products. The WTP for certification (71%) is higher than for labeling (64%), suggesting that consumers place greater value on the additional credibility and assurance provided by certification. The findings are consistent with the results reported by Hossain et al. [1]. The high percentage of positive responses in both cases reflects a growing consumer demand for transparency, safety, and quality in food products. This trend indicates a market opportunity for producers and retailers to differentiate their products through labeling and certification. These findings highlight the potential for developing policies and market mechanisms that support and promote food labeling and certification initiatives. Encouraging these practices can enhance food safety standards and consumer confidence in the market.

The study also identified the factors that influence consumers’ WTP labeling and certification. Two separate binary logit models were employed for the WTP of labeling and certification, respectively. Table 2 reveals that several family and personal characteristics influence consumers’ WTP for labeling and certification. Results show that respondents’ household income had a positive and significant effect on their WTP for both labeling and certification. The MEs indicate that if household income increased by one unit, the probability of consumers’ WTP for labeling and certification increased by 20.9% and 8.7%, respectively. The positive and significant effect of household income on WTP labeling and certification aligns with economic theory, which posits that higher income levels increase consumers’ ability to purchase premium products. In the context of this study, higher income households are more likely to afford the additional costs associated with labeled and certified chicken meat [40]. Products with attributes such as labeling and certification typically command higher prices due to the additional costs involved in ensuring compliance with safety and quality standards. Consumers with higher incomes may place greater value on these attributes as they seek quality assurance and safety in their food purchases. This WTP premium reflects a demand for transparency and trust in the food supply chain.

The educational level of respondents affected their WTP for labeling and certification positively and significantly. Results illustrate that a one-unit increase in the education level of consumers would raise the probability of consumers WTP labeling and certification by 1.7% and 2%, respectively. Higher educational levels often correlate with increased awareness and knowledge about various issues, including food safety, nutrition, and health. Educated consumers are more likely to understand the importance of labeling and certification as indicators of product quality and safety standards. This heightened awareness translates into a greater willingness to invest in products that offer these assurances [32].

**Table 2.** Results of logistic regression of WTP for labeling and certification of chicken meat.

Explanatory variables	WTP for labelling		WTP for certification	
	Coefficient	ME	Coefficient	ME
Income (BDT)	0.859*** (0.236)	0.209*** (0.057)	0.531** (0.269)	0.087** (0.043)
Age (years)	−0.021 (0.016)	−0.005 (0.004)	−0.011 (0.016)	−0.002 (0.003)
Education (years of schooling)	0.069*** (0.024)	0.017*** (0.006)	0.121*** (0.027)	0.020*** (0.004)
Marital status (dummy)	−0.419 (0.421)	−-0.102 (0.102)	−0.339 (0.465)	−0.056 (0.077)
Dependency ratio	−0.244** (0.120)	−0.059** (0.029)	−0.041 (0.123)	-0.007 (0.020)
Market distance (km)	−0.412 (0.305)	−0.100 (0.074)	−0.811** (0.344)	−0.133** (0.055)
Health perception	0.049*** (0.013)	0.012*** (0.003)	0.064*** (0.014)	0.011*** (0.002)
Environment perception	0.037** (0.018)	0.009** (0.004)	0.010 (0.012)	0.002 (0.002)
Constant	−13.323*** (2.828)		−9.23*** (2.912)	
Number of observations	300		300	
LR χ2(8)	100.67***		90.42***	
Pseudo *R*^2^	0.2438		0.2515	
Log pseudolikelihood	−156.108		−134.531	

Note: *** and ** indicate the significance level at 1% and 5% respectively. Values in parentheses indicate standard errors.

The dependency ratio of the households had a significant adverse effect on the consumers’ WTP for labeling but not on the preference of certification. The ME showed that when households’ dependency ratio increased by one unit, the probability of consumers’ WTP for labeling decreased by 5.9%. The dependency ratio is the proportion of non-working (dependent) members to working (earning) members within a household. A higher dependency ratio suggests that a larger portion of the household comprises dependents, such as children and the elderly, relative to earners. This ratio impacts the overall financial flexibility and discretionary spending capacity of the household. This decline in WTP indicates that households with more dependent members than earning members would pay for labeling less than their counterparts, which is in line with the findings of [33]. This unwillingness might be influenced by the anticipated price increase. Such households might prioritize essential expenditures over premium food attributes like labeling, especially if they perceive the additional cost as non-essential.

Contrarily, distance from the market had a significant negative effect on the consumers’ WTP for certification but not on the preference of labeling. When market distance increased by one unit, the probability of WTP for certification of the consumers decreased by 13.3%. Consumers living far from the market might have additional transportation costs, which could be the reason behind this result. Certification often implies a higher price point due to the rigorous processes involved. When consumers face additional transportation costs, the total expense of purchasing certified products might become prohibitive. Labeling, while valuable, might be perceived as less critical or more ubiquitous compared to certification. Hence, the financial burden of extra transportation costs may not significantly encourage consumers to purchase labeled products.

Results also revealed that consumers’ health perceptions had a significant positive influence on their WTP for both labeling and certification. Health perception refers to consumers’ beliefs and attitudes about the health benefits and safety of the food they eat. The ME results revealed that a unit increase in consumers’ health perception would increase the probability of their WTP being labeled and certified by 1.2% and 1.1%, respectively. It indicates that the more consumers perceive chicken meat as healthy, the more willing they are to pay for labeling and certification. As consumers become more health-conscious, they seek assurance that the food they consume meets high safety and quality standards. Labeling and certification serve as indicators of these standards, providing consumers with confidence in their food choices. Health-conscious consumers prioritize food attributes that ensure safety and nutritional quality. This preference translates into a higher WTP for products that are labeled and certified, as these attributes are perceived to enhance food safety and health benefits. A similar finding was reported by Mohamed et al. [41].

The environmental perception of the consumers had a significant positive effect on their WTP for labeling but not on WTP for certification. Environmental perception refers to consumers’ awareness and attitudes towards the environmental impact of their consumption choices. The ME indicates that one one-unit increase in consumers’ environmental perception would increase their probability of paying for labeling by 0.9%. It implies that environmentally conscious consumers would pay more for labeling and the certification of chicken meat. This finding is in accordance with the study of [42]. Environmentally conscious consumers are more likely to prioritize sustainability and ecological impact when making purchasing decisions. These consumers seek products that align with their environmental values, often favoring those that indicate eco-friendly practices and sustainability. Labels can provide specific information about the environmental impact, such as organic certification, sustainable farming practices, and eco-friendly packaging. This transparency resonates with environmentally conscious consumers.

## Conclusion

The findings reveal that a significant portion of consumers are willing to pay extra for these attributes, emphasizing the importance of food safety and quality assurance in the poultry industry. Based on this study, labeling and certification of chicken should be implemented to ensure safe chicken meat since consumers are willing to pay a premium price. The study underscores the potential benefits of implementing robust labeling and certification systems in Bangladesh’s poultry sector. Such measures could significantly enhance food safety, boost consumer trust, and improve public health outcomes. By addressing the factors influencing WTP, policymakers and industry stakeholders can develop targeted strategies to foster consumer confidence and drive industry growth, ultimately contributing to the broader economy. This research provides valuable insights into the dynamics of consumer behavior in the context of food safety and offers a framework for future policy development in the poultry industry.

## List of Abbreviations

CVM, Contingent Valuation Method;WTP, Willingness to pay.
